# Supplementary material for: Droplet Digital PCR Enhances Sensitivity of Canine Distemper Virus Detection
Source: Viruses. 2024 Oct 31;16(11):1720. doi: 10.3390/v16111720 (PMC11598849; doi:10.3390/v16111720)
Supplement: Supplementary file 1 [file viruses-16-01720-s001.zip › viruses-3235773-supplementary.pdf]

S1. Canine distemper virus infection in domestic dogs. HE and LFB stainings at three progessive stages (normal, sub-acute and chronic), demyelinating changes in white matter, and CDV immunoreactive particle deposition is shown in each stage.

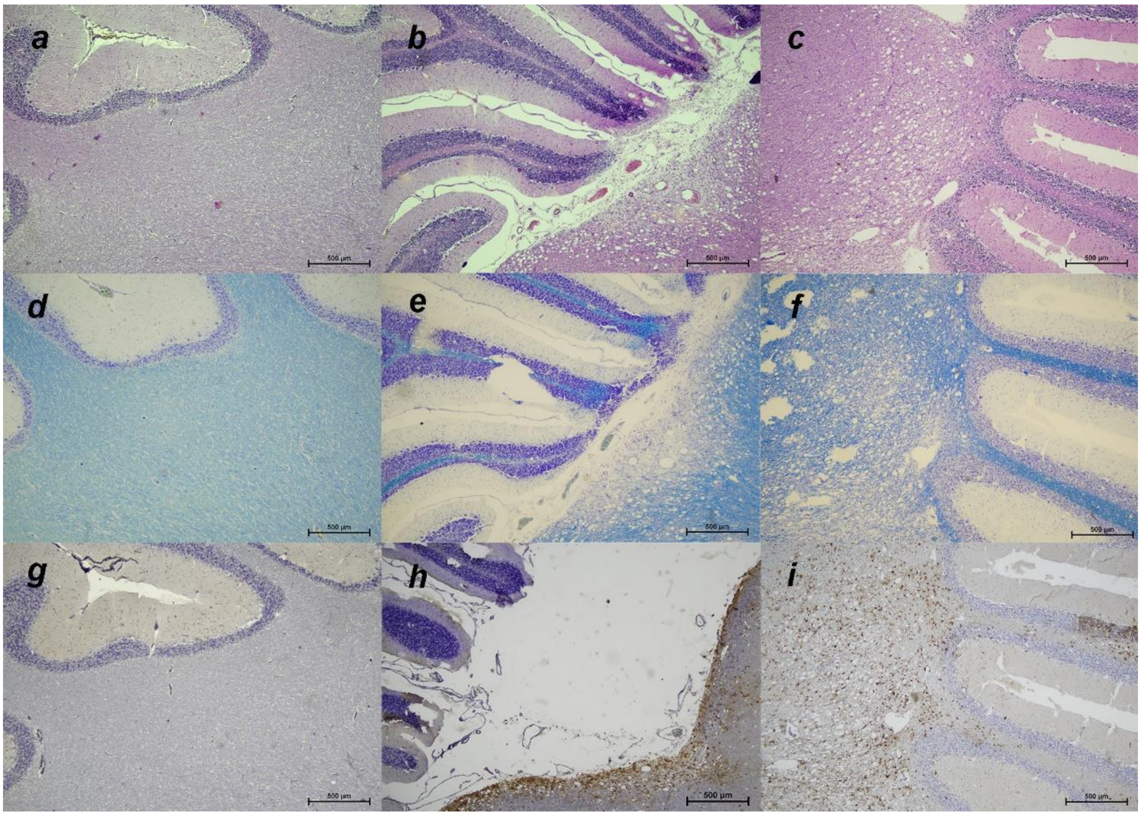

Figure S1. **(a, d, g)** Demyelination is not detected. Dog of Control group. **(a)** HE; **(d)** LFB; **(g)** Immunohistochemistry against Canine Distemper Virus (CDV) ribonucleoproteins. Scale=500µm. **(b, e, h)** Demyelination is apparent. Dog of Subacute (2) group. **(b)** HE; **(e)** LFB; **(h)** Immunohistochemistry against Canine Distemper Virus (CDV) ribonucleoproteins. Scale=500µm. **(c, f, i)** Demyelination is severe. Dog of Chronic group. **(c)** HE; **(f)** LFB; **(i)** Immunohistochemistry against Canine Distemper Virus (CDV) ribonucleoproteins. Scale=500µm.
